# Microwave-Assisted Freeze–Drying: Impact of Microwave Radiation on the Quality of High-Concentration Antibody Formulations

**DOI:** 10.3390/pharmaceutics15122783

**Published:** 2023-12-15

**Authors:** Nicole Härdter, Raimund Geidobler, Ingo Presser, Gerhard Winter

**Affiliations:** 1 Department of Pharmacy, Pharmaceutical Technology and Biopharmaceutics, Ludwig-Maximilians-Universität München, 81377 Munich, Germany; 2 Boehringer Ingelheim Pharma GmbH & Co. KG, Pharmaceutical Development Biologicals, 88397 Biberach an der Riß, Germany

**Keywords:** freeze-drying, lyophilization, microwave, protein, monoclonal antibody, stability, aggregation

## Abstract

Microwave-assisted freeze-drying (MFD) offers significant time savings compared to conventional freeze-drying (CFD). While a few studies have investigated the stability of biopharmaceuticals with low protein concentrations after MFD and storage, the impact of MFD on high-concentration monoclonal antibody (mAb) formulations remains unclear. In this study, we systematically examined the effect of protein concentration in MFD and assessed protein stability following MFD, CFD, and subsequent storage using seven protein formulations with various stabilizers and concentrations. We demonstrated that microwaves directly interact with the active pharmaceutical ingredient (API), leading to decreased physical stability, specifically aggregation, in high-concentration antibody formulations. Furthermore, typically used sugar:protein ratios from CFD were insufficient for stabilizing mAbs when applying microwaves. We identified the intermediate drying phase as the most critical for particle formation, and cooling the samples provided some protection for the mAb. Our findings suggest that MFD technology may not be universally applicable to formulations well tested in CFD and could be particularly beneficial for formulations with low API concentrations requiring substantial amounts of glass-forming excipients, such as vaccines and RNA-based products.

## 1. Introduction

Although antibody therapeutics are now preferably formulated as liquid formulations, offering greater flexibility for patients, such as self-administration through pen devices [1,2], lyophilization remains the standard method when a particular molecule is facing stability issues [3]. Numerous reviews have been provided on the rational design of robust and optimized freeze-drying processes [4,5,6,7], as well as ideas for speeding up the typically lengthy process [8,9,10,11]. More recently, microwave-assisted freeze-drying (MFD) has gained attention due to its potential for significant time savings while maintaining the product quality of probiotics [12], vaccines, and proteins [13,14] and, more specifically, monoclonal antibodies (mAbs) [15,16,17]. While heat transfer in conventional freeze-drying (CFD) is primarily limited to convection, with some conduction and radiation, microwaves directly interact with the dipolar molecules of the formulation [18]. Energy is mainly transferred due to dipole rotation for permanent dipoles, i.e., in most biological materials [18]. The dielectric properties of a pharmaceutical formulation strongly depend on the concentration of buffer salts and disaccharides, typically used for cryo- and lyoprotection, as well as the amount of unfrozen water. Residual water great affects heat transfer because of the much higher effective loss factor of water compared to ice [19]. We hypothesized that microwaves excite the unfrozen water, and this causes the glass transition temperature T_g_′ to increase during drying [17]. As a result, drying processes become more robust and can be conducted very fast without impairing cake appearance. Interested readers should refer to works [19,20,21] for more information on microwave heating.

We recently introduced a new MFD setup that overcomes the drawbacks of previous machines, as it enables in-chamber freezing and stoppering [17]. This setup combines the advantages of a conventional lyophilizer, which was designed with good manufacturing practice (GMP) processes in mind, with microwave radiation. It employs flat, solid microwave modules that can be flexibly incorporated into the process. For details on the new setup, readers are referred to [17]. Additionally, we assessed mAb stability following MFD and found it to be comparable to mAb stability following CFD. Recent studies have focused on low-concentration protein formulations [13,14,15], with 50 mg/mL being the highest mAb concentration investigated [16]. However, in recent years, high-concentration antibody formulations have become immensely popular and successful [22], with 46 approved products ≥ 100 mg/mL in the US [1]. One of the major challenges in developing these formulations is protein aggregation, as it can increase at higher concentrations [23]. 

This work aims to explore the microwave-assisted freeze-drying of such high-concentration antibody formulations. We sequentially replaced sugar with antibodies to study their effect on the MFD process and protein stability. While drying times varied slightly, we observed reduced stability in the mAb when less stabilizing sugar was present in the formulation. These results prompted us to compare the stability profiles of high-concentration formulations directly after production with MFD and CFD, and after storage for up to six months at 4 °C, 25 °C, and 40 °C. When we found increased aggregate formation following MFD, we tried to identify the critical timeframe for degradation during the MFD process. Further studies using a microwave oven were then carried out to investigate whether microwave radiation directly interacts with the mAb, and how different levels of molecular mobility in the cake may affect this.

## 2. Materials and Methods

### 2.1. Proteins and Chemicals

In this study, two monoclonal IgG type-1 antibodies (mAbs) were used: one sourced from the laboratory’s stock (LMU1, Munich, Germany), and the other (LMU2) generously provided by Boehringer Ingelheim Pharma GmbH & Co. KG (Ingelheim am Rhein, Germany). Further, G-CSF (filgrastim) was used as a model protein. L-histidine (cell culture reagent) and L-histidine monohydrochloride monohydrate (99% purity) were purchased from Alfa Aesar (Ward Hill, MA, USA). EMPROVE^®^ exp sucrose, EMPROVE^®^ exp di-sodium hydrogen phosphate dihydrate, EMPROVE^®^ bio sodium chloride, sodium citrate dihydrate (≥99.0%), and L-methionine were purchased from Merck KGaA (Darmstadt, Germany). D(+)-trehalose dihydrate (97.0–102.0% purity) Ph. Eur., NF certified, and D(-)-mannitol (97.0–102.0% purity) Ph. Eur., USP certified were purchased from VWR International (Radnor, PA, USA). Sodium dihydrogen phosphate dihydrate (99%) was purchased from Grüssing GmbH (Filsum, Germany). Trizma^®^ base and Trizma^®^ hydrochloride (both in BioXtra grade), anhydrous citric acid BioUltra grade (≥99.5%), and sodium azide (≥99.5%) were purchased from Sigma Aldrich (Burlington, MA, USA). Super Refined™ Polysorbate 20-LQ-(MH) was purchased from Croda (Edison, NJ, USA). All solutions were prepared using ultrapure water from a Sartorius Lab Instruments GmbH Arium^®^ system (Goettingen, Germany).

### 2.2. Preparation of the Formulations

We used seven different verum formulations (Table 1). For F1–F5, we dialyzed and concentrated the mAb bulk solution using a Minimate™ Tangential Flow Filtration (TFF) capsule (MWCO 30 kDa; Pall Corporation, New York, NY, USA). A sevenfold excess of 10 mM histidine buffer (pH 5.5) was used for thorough dialysis, resulting in a final buffer mixture that contained 10 mM histidine and 0.04% (*w*/*v*) polysorbate 20. We determined the mAb concentration using a Nanodrop 2000 UV spectrophotometer (Thermo Fisher Scientific, Waltham, MA, USA) at 280 nm, based on the molar extinction coefficient. Excipient stock solutions were prepared in 10 mM histidine buffer and combined with the protein solution according to the target composition (Table 1). Formulation F6 was already provided in the final composition. For F7, the protein bulk solution underwent buffer exchange at 2–8 °C using Slide-A-Lyzer™ 2000 molecular weight cut-off dialysis cassettes (Thermo Fisher Scientific, Waltham, MA, USA). The sample-to-buffer ratio was 1:100, and buffer exchange was performed after 3 and 6 h, following dialysis overnight. All excipients were already added to the dialysis buffer, except for the surfactant, which was introduced after dialysis as a stock solution in 20 mM sodium citrate buffer. Following this, protein concentration was determined with a Nanodrop 2000 UV spectrophotometer (Thermo Fisher Scientific, Waltham, MA, USA) at 280 nm, and the formulation buffer was combined with the dialyzed protein solution. All formulations were sterile-filtered prior to lyophilization using 0.22 µm Sartolab^®^ RF polyether sulfone vacuum filtration units (Sartorius AG, Goettingen, Germany).

### 2.3. Freeze-Drying Process

Four distinct lyophilization cycle protocols were used (Table 2), with references to the respective processes provided in the text. For all processes, formulations were poured into 63 10R FIOLAX vials (MGlas AG, Muennerstadt, Germany) and placed on the middle of the shelf in a hexagonal array. Shelves were then cooled to −50 °C and held at the respective temperature until the product was completely frozen. For formulation F7, an additional annealing step was performed at −20 °C for 4 h, to enable the crystallization of mannitol.

Processes P1, P3, and P4 were conducted using a laboratory-scale freeze-dryer from OPTIMA Pharma GmbH (Schwäbisch Hall, Germany), which was equipped with flat, emitting semiconductor microwave modules. The vials were organized in a hexagonal pattern (180 mm × 190 mm) at the center of a shelf (486 mm × 440 mm). The microwave modules were attached to the underside of the shelf above the vials, covering an antenna area of approximately 26 cm × 26 cm. The modules were operated at 2.43–2.48 GHz and exhibited exceptional mechanical stability, which enabled the stoppering of the vials following the drying process. Experiments were conducted in the machine manufacturer’s technical workshop. As thermocouples and resistance temperature detectors would not work in the given electromagnetic environment, fiberoptic temperature sensors (Weidmann Technologies Deutschland GmbH, Dresden, Germany) were utilized for product temperature recording. A mass spectrometer (Pfeiffer Vacuum GmbH, Asslar, Germany) was employed, in conjunction with comparative pressure measurement via a Pirani and capacitance gauge, to monitor the drying process. Process P1 was designed to adhere to the typical format of primary and secondary drying steps, enabling a detailed study of protein concentration effects on MFD processes. Processes P3 and P4 aimed to compete with aggressive CFD processes and were used to investigate the impact of the duration of microwave radiation on highly concentrated mAb formulations.

Process P2 was used to apply a comparable thermal history to CFD samples, as for those dried with microwave assistance. It was performed either on an FTS LyoStar™ 3 (SP Scientific, Stone Ridge, NY, USA) or a Christ ε2-6D (Martin Christ, Osterode am Harz, Germany) laboratory-scale freeze-dryer. 

Once the drying processes were completed, the vials were stoppered under vacuum within the chamber of the lyophilizers in a nitrogen atmosphere, followed by capping with Flip-Off^®^ seals (West Pharmaceutical Services, Inc., Exton, PA, USA). Subsequently, they were stored at 2–8 °C upon further processing.

### 2.4. Karl–Fischer Titration

The lyophilizates’ residual moisture content was measured using coulometric Karl–Fischer titration. In a controlled-humidity environment (relative humidity (rH) < 10%), the lyophilized cakes were carefully crushed, and portions weighing 40–90 mg were transferred into 2R vials. These samples were then heated at 100 °C in an oven, and the extracted water was carried to the coulometric titration cell using a dry gas flow (Aqua 40.00 Vario Plus, ECH Elektrochemie Halle GmbH, Halle (Saale), Germany). The Apura^®^ water standard oven 1% (Merck KGaA, Darmstadt, Germany) was used in triplicate to confirm the equipment’s performance before analyzing the samples. The relative residual moisture content was calculated considering the cake mass (*w*/*w*).

### 2.5. Brunauer–Emmet–Teller (BET) Krypton Gas Adsorption

The Brunauer–Emmet–Teller (BET) method was employed to measure the specific surface area of the lyophilizates. Under controlled-humidity conditions (relative humidity < 10%), at least 100 mg of gently crushed samples was placed into 9 mm sample cells. The sample cells were cooled in a liquid nitrogen bath (77 K), and quantity of absorbed krypton gas was measured with an Autosorb 1 (Quantachrome, Boynton Beach, FL, USA). Krypton adsorption was determined over a p/p_0_ ratio of 0.05–0.30 (11-point BET). An outgassing procedure was carried out at ambient temperature for a minimum of 2 h prior to the analysis. The Autosorb 1.55 software was used to calculate the specific surface area, applying the multipoint BET method fit.

### 2.6. Scanning Electron Microscopy (SEM)

The morphology of the lyophilizates was investigated using a Helios NanoLab G3 UC (FEI, Hillsboro, OR, USA) scanning electron microscope (SEM) at an acceleration voltage of 2 kV. Fragments from the top and bottom layers of the cakes were extracted in a glove box with a relative humidity of less than 10%. The samples were then sputtered with a 10 nm carbon layer using a CCU-010 HV sputterer (Safematic GmbH, Zizers, Switzerland). Images were captured at 175-fold magnification.

### 2.7. Experiments with the Microwave Oven

A Bosch HMT84M421 microwave oven (Robert Bosch Hausgeräte GmbH, München, Germany) was used to study the effect of microwave radiation on mAb stability. Prior to the experiments, flip-off seals were removed, and a single vial was positioned at the center of the rotating plate. A stainless steel cylinder, measuring approximately 5 cm × 3 cm, was pre-chilled at −70 °C for one hour and subsequently used intermittently to cool the samples during irradiation. Microwave power levels of 180 W, 360 W, and 600 W were applied for specific time intervals. Afterward, the samples were reconstituted and subjected to analysis. To monitor the sample temperature, an Ebro TLC 750i thermometer (Xylem Analytics Germany GmbH, Weilheim, Germany) was used. To discern the effects of microwave radiation on the mAb from mere sample heating, the samples were placed in a Heraeus UT 20P drying cabinet (Thermo Fisher Scientific, Waltham, MA, USA).

### 2.8. Reconstitution of the Lyophilizates

The lyophilizates were reconstituted via the addition of ultrapure water. The necessary volume was individually determined for each formulation to correspond with the volume of water removed during the lyophilization process.

### 2.9. Size-Exclusion Chromatography (SEC)

A Thermo Scientific™ Dionex™ UltiMate™ 3000 UHPLC system was used in conjunction with a VWD-3400RS UV/Vis absorbance detection unit from Thermo Fisher Scientific (Waltham, MA, USA) to measure monomer yield and protein aggregates. First, 100 µg of LMU1 and LMU2 was injected onto a TSKgel G3000SWxl, 7.8 × 300 mm, 5 µm column (Tosoh Bioscience, Tokyo, Japan). The running buffer consisted of 100 mM sodium phosphate, 300 mM sodium chloride, and 0.05% (*w*/*v*) sodium azide at pH 7.0. For F7, 15 µg of G-CSF were injected onto a Superdex™ 75 Increase 10/300 GL, 10 × 300 mm column (GE Healthcare Bio-Sciences AB, Uppsala, Sweden). The mobile phase was composed of 100 mM sodium phosphate and 0.05% (*w*/*v*) sodium azide at pH 7.0. Both columns were operated at a flow rate of 1 mL/min. Absorption at 280 nm was used to detect elution, and the resulting chromatograms were integrated using Chromeleon™ 7.2.7 software (Thermo Fisher Scientific, Waltham, MA, USA). The monomer yield relative to the amount of monomer before freeze-drying the specific formulations was calculated. The method described in [24] was used to determine the relative number of high-molecular-weight species (HMWS).

### 2.10. Cation-Exchange Chromatography (IEX)

A Thermo Scientific™ Dionex™ UltiMate™ 3000 UHPLC system, featuring a VWD-3400RS UV/Vis absorbance detector and equipped with a ProPac™ WCX-10G BioLC™ analytical column (4 × 250 mm) together with a ProPac™ WCX-10G BioLC™ guard column (4 × 50 mm), all from Thermo Fisher Scientific (Waltham, MA, USA), was utilized to examine the chemical stability of LMU1. Mobile phase A was composed of 20 mM TRIS (pH 8.0), while mobile phase B consisted of 20 mM TRIS and 300 mM sodium chloride (pH 8.0). A linear salt gradient mode was used for elution, ranging from 0% B to 20% B over 30 min at a flow rate of 1 mL/min. Prior to analysis, samples were diluted 1:100 using mobile phase A, and the injection volume was 10 µL or 100 µL depending on the mAb concentration. Detection of elution occurred at 280 nm, and chromatogram integration was carried out using Chromeleon™ 7.2.7 software (Thermo Fisher Scientific, Waltham, MA, USA). The integrated chromatograms were categorized into three components: the main peak, acidic variants associated with each peak that eluted prior to the main peak, and basic variants linked to each peak that eluted after the main peak.

### 2.11. Flow Imaging Microscopy

The analysis of subvisible particle formation was conducted using a FlowCam 8100 (Fluid Imaging Technologies, Inc., Scarborough, ME, USA). The instrument was outfitted with a 10× magnification flow cell (80 µm × 700 µm) and was operated via VisualSpreadsheet^®^ 4.7.6 software. A sample of 150 µL was analyzed at a flow rate of 0.15 mL/min, with particle images captured at an automatic frame rate of 28 frames/second. Parameters for particle identification were 3 µm distance to the nearest neighbor and particle thresholds of 13 and 10 for dark and light pixels, respectively. Particle sizes were presented as equivalent spherical diameters.

## 3. Results and Discussion

### 3.1. Substitution of Sugar by an Antibody

From CFD, it is well established that increasing protein concentrations lead to more robust drying processes due to a rise in the difference between the glass transition temperature (T_g_′) and collapse temperature (T_c_) [25]. Consequently, the occurrence of collapse becomes less likely; however, it is important to consider the substantial dry-layer resistances to mass flow associated with high protein concentrations. However, the relationship between microwave-assisted freeze-drying processes and protein concentrations remains unclear. Recent studies have demonstrated that increasing the solute concentrations of stabilizers, such as sucrose and trehalose, results in enhanced dielectric heating [13,17]. To further investigate the effect of protein concentration in microwave-assisted drying processes, we gradually substituted sucrose with mAb (F1–F4, Table 1) and applied drying process P1 (Table 2). The overall solid content in all these samples was kept constant at ca. 9.0% (*w*/*v*) = 90 mg/mL. We observed that the drying time increased only slightly with higher mAb concentrations. With microwave assistance, F1 was dried within 28.5 h, while F2, F3, and F4 took 28.8 h, 29.5 h, and 30.3 h, respectively. 

**Figure 1:**
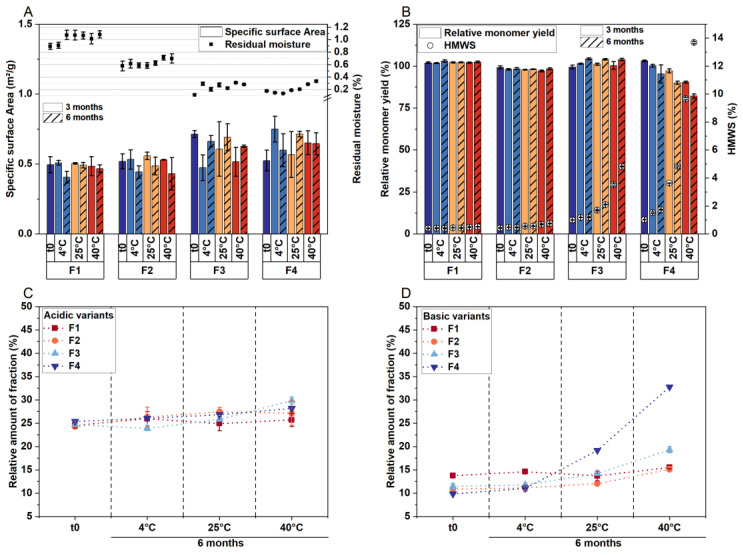
The solid-state properties of the lyophilizates and storage stability of LMU1 when sugar was subsequently replaced with mAb. Samples were analyzed after MFD (t0) and storage at 4 °C, 25 °C, and 40 °C over 6 months. (**A**) Specific surface area (bars) and residual moisture (symbols). The relative monomer yields (bars) and percentages of soluble aggregates (HMWS, symbols) from SEC are shown in (**B**). (**C**) The relative number of acidic and (**D**) basic variants from IEX. All values are means (*n* = 3) ± standard deviation.

The lyophilizates appeared elegant on a macroscopic scale and scanning electron microscopy revealed a cellular pore structure for F2–F4 on a microscopic scale, whereas F1 exhibited microcollapse (Figure S1). Due to the low T_g_′ of low-concentrated mAb formulations in combination with sucrose, microcollapse may not be avoided with harsh drying conditions regardless of the application of microwaves [26], and we likewise observed microcollapse for F1 following CFD [27]. For low-concentration protein formulations, T_g_′ and T_c_ are interchangeable [25]. Therefore, when the product temperature during drying exceeds the glass transition temperature for such formulations, the microstructure of the cake undergoes viscous flow and eventually collapses. The cake morphology corresponded with the observed specific surface areas after lyophilization, and stability study data suggest that it was maintained throughout the study (Figure 1A). Moreover, the residual moisture in the lyophilizates correlated with the sucrose concentration, i.e., samples became drier when the protein content was increased at the cost of the sugar (Figure 1A).

Regarding the physical stability of the mAb, aggregate formation increased with decreasing sucrose concentrations, both immediately after lyophilization and after six months of storage (Figure 1B). The same trend was observed for the chemical stability of LMU1 (Figure 1C–D), with F4 showing the highest number of basic variants after storage at 40 °C. An increase in basic species could be attributed to various modifications, including oxidation, succinimide formation, or disulfide-mediated changes [28]. Moreover, when the formulation contained less stabilizing sugar, the water replacement during the drying process was inadequate. Consequently, the protein was not stabilized in its native state, leading to the formation of aggregates. Past research has shown that aggregates of an IgG1 have a high affinity for cation-exchange columns and, as a result, they elute in the basic variant region in IEX [28]. Therefore, it can be inferred that aggregate formation in formulations with less stabilizing sugar and a concurrent increase in basic species are related to each other. Previous research has indicated that the sugar:protein ratio is crucial for protein stabilization during drying and storage [28,29]. Consequently, it appears that the reduced protein stability with a decreasing sugar:protein ratio is not due to microwave application but is generally related to less protection against stresses during the lyophilization process. For F1, the molar ratio of disaccharide to protein was significantly above the proposed proportion [29], at approximately 3500:1, while it was 125:1 for F4.

### 3.2. Comparison of Critical Quality Attributes of a Highly Concentrated mAb Formulation following MFD and CFD

**Figure 2:**
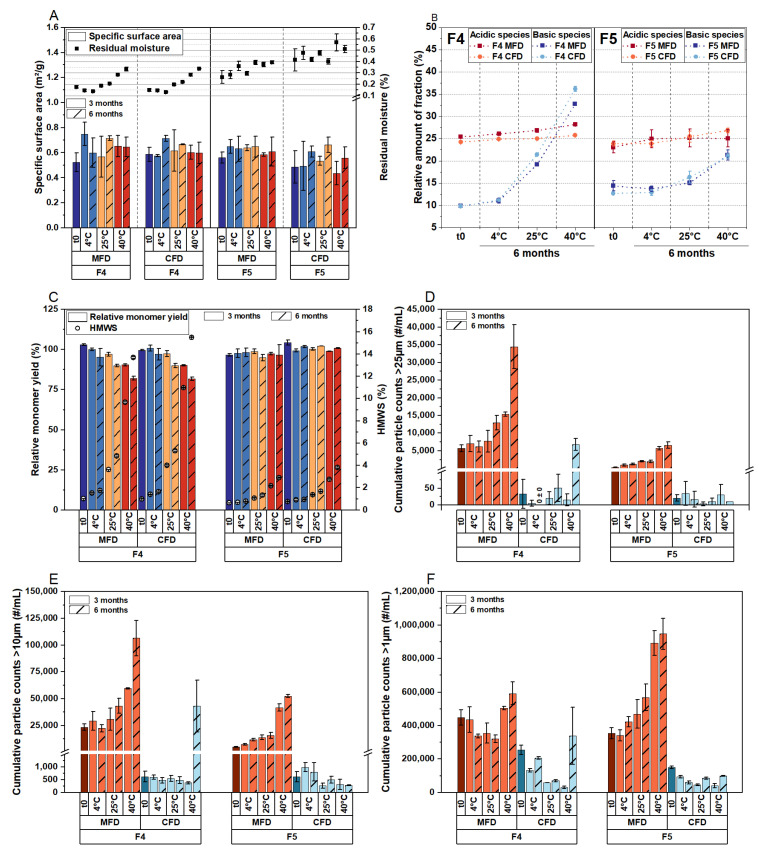
The effect of the drying mechanism on critical quality attributes of highly concentrated LMU1 formulations. Following MFD and CFD (t0), the lyophilizates were stored at 4 °C, 25 °C, and 40 °C for 6 months. (**A**) The specific surface area (bars) and residual moisture (symbols) of the cakes. (**B**) The relative number of acidic and basic variants for F4 (left) and F5 (right) from IEX. (**C**) The relative monomer yield and the relative number of high-molecular-weight species (HMWS) was determined using SEC. Subvisible particles (SvP) detected with flow imaging microscopy: (**D**) >25 μm, (**E**) >10 µm, and (**F**) >1 μm. All values are means (*n* = 3) ± standard deviation. SvP measurements were conducted in technical duplicates.

Based on the previous results, we aimed to directly compare the stability profiles of high-concentration mAb formulations following MFD and CFD. Consequently, we selected formulation F4, representing a worst-case scenario in terms of stabilizer concentration, and F5, which comprises a typically used sugar:protein ratio (350:1) sufficient for stabilizing monoclonal antibodies [29]. Furthermore, F5 comprises the same proportion of lyoprotectant to mAb as F3, but with twice the overall solute content. With microwave assistance, F5 was dried within 29.9 h, while it took 59.6 h with CFD. Moreover, it took 56.3 h to lyophilize F4 without microwaves, compared to 30.3 h using MFD. Samples were analyzed immediately after lyophilization (Process P1, Table 2) and following storage at 4 °C, 25 °C, and 40 °C over 6 months. The results are shown in Figure 2.

The solid-state properties of the lyophilizates were very similar, irrespective of whether MFD or CFD was applied (Figure 2A). However, given that the same drying protocol (Process P1, Table 2) was used for both MFD and CFD, and the formulations consisted of high protein concentrations, the drying process was anticipated to be highly robust (i.e., with a high T_c_). When comparing the relative number of acidic and basic variants, we observed no relevant differences between the two drying protocols (Figure 2B). The monomer yields and aggregate formations exhibited the same trends during the stability study (Figure 2C), with F4 demonstrating a lower capability in stabilizing the mAb compared to F5. However, this observation was independent of the application of microwave radiation.

Notably, subvisible particle analysis revealed increased particle formation following MFD compared to CFD across all size ranges (Figure 2D–F). Previous studies did not report this phenomenon, but most cases involved low concentrations [13,14,15,17] up to 50 mg/mL mAb [16]. To further investigate this observation, we sought to identify the root cause for the formation of subvisible particles following MFD.

### 3.3. Effect of Thermal History and Investigation of Two Other Proteins in MFD

Considering these findings, we aimed to determine if the particle formation for LMU1 is a consequence of higher product temperatures during the MFD process compared to CFD. To investigate this, we conducted a single-step CFD cycle (Process P2, Table 2) using formulation F5 to simulate the thermal history during the corresponding MFD process. The respective readouts are presented in Figure S2. The residual moisture was found to be comparable following both drying processes (0.34% ± 0.02% after CFD and 0.23% ± 0.06% following MFD). Subvisible particle counts (given in #/mL cumulatively) were detected using flow imaging microscopy. We observed low subvisible particle counts after the aggressive CFD cycle with 10 ± 11, 110 ± 55, and 3444 ± 1017 for particles ≥25 µm, ≥10 µm, and ≥1 µm in size, respectively. After 7 months of storage at 40 °C, the subvisible particle counts were close to the initial amounts with 13 ± 13, 64 ± 35, and 4658 ± 428 for the respective sizes. Consequently, we concluded that high product temperatures during drying are not responsible for particle formation following MFD.

**Figure 3:**
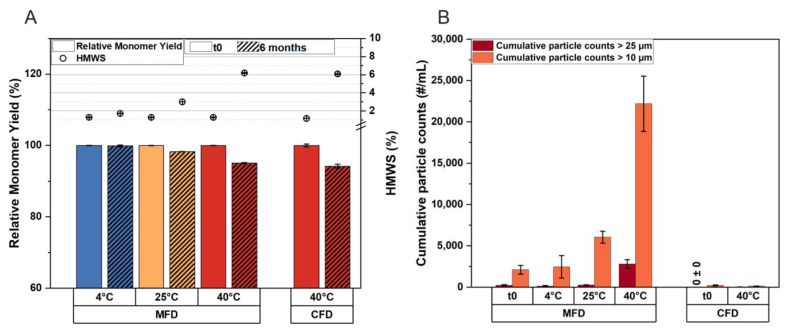
Physical stability of LMU2 (formulation F6) following MFD and CFD. Samples were analyzed after lyophilization (t0) and storage at 4 °C, 25 °C, and 40 °C (MFD samples) and 40 °C (CFD samples). (**A**) The relative monomer yield and the relative number of high-molecular-weight species (HMWS). (**B**) Subvisible protein aggregates. All values are means (*n* = 3) ± standard deviation. Subvisible particle measurements were conducted in technical duplicates.

Next, we examined another mAb (Formulation F6, Table 1) to assess whether particle formation is specific to LMU1. To compare stability profiles, LMU2 was dried with and without microwaves using process P1. For F6, the molar sugar:protein ratio was approximately 360:1. Samples were analyzed immediately after lyophilization and after storage. Again, the residual moisture was found to be comparable following the drying processes (0.18% ± 0.01% after CFD and 0.20% ± 0.15% following MFD). No differences were detected in the monomer yield and the formation of high-molecular-weight species in SEC (Figure 3A). However, as with LMU1, the subvisible particle counts revealed a significant increase in protein aggregation following MFD compared to CFD (Figure 3B).

In a published study, we had investigated the stability of an IgG1 at low concentration in different formulations after MFD and storage. We had observed similar stability profiles following MFD and CFD [17]. These findings contrast with the results from this study on high-concentration antibody formulations, prompting us to examine the quality of another low-concentration protein, G-CSF (formulation F7) after MFD. Following the MFD process (Process P3, Table 2), the monomer yield was 96.70% ± 0.70%. Protein aggregates detected with SEC (0.27% ± 0.30% high-molecular-weight species) and flow imaging microscopy (0 ± 0 > 25 µm, 45 ± 33 > 10 µm, and 1128 ± 498 > 1 µm) were low. Based on these data, we consider that aggregation triggered by microwave radiation is directly related to protein concentration. Since microwaves directly interact with dipolar structures [21], we conclude that electromagnetic radiation excites not only the excipients but also the protein. As a result, the higher the protein concentration in the formulation, the greater the likelihood of inducing damage.

### 3.4. The Critical Timeframe That Leads to Protein Aggregation during MFD

**Figure 4:**
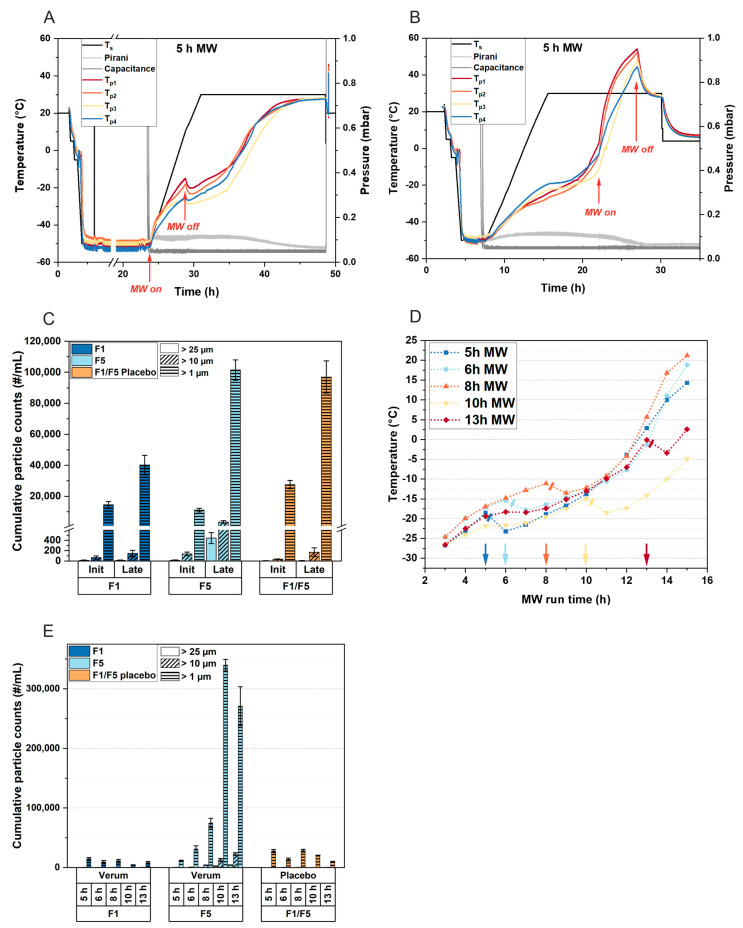
The impact of the microwave run time on protein aggregation during MFD. (**A**) Graphical overview of the lyophilization process readouts for P3. Microwave radiation was started immediately after the desired vacuum for primary drying was established and ran for 5 h. T_s_ denotes the shelf temperature; the chamber pressure is monitored via a Pirani gauge (Pirani) and capacitance gauge (Capacitance); T_p_ is the reading from the fiberoptic temperature sensors. (**B**) Process readouts for P3 when microwave radiation was applied for 5 h toward the end of the process. (**C**) Comparison of subvisible particle formation in the F1, F5, and placebo formulations, as detected via flow imaging microscopy, when microwave radiation was applied during the initial 5 h of drying (init) and for 5 h later in the process (late), using process P3. (**D**) Product temperature profiles recorded for P3 and P4 with the different microwave module run times. The arrows represent the switch off of microwave radiation. All temperature sensors shown in the process graphs (**A**,**B**,**D**) were placed in formulation F5. (**E**) Subvisible particle formation in the F1, F5, and placebo formulations when subjected to increasing microwave run times. The reported numbers of subvisible particles are means (*n* = 3 and technical duplicates per vial) ± standard deviation. MW, microwave irradiation.

To investigate the mechanism of particle formation in MFD processes, we used formulations F1 and F5 and the corresponding placebo. We temporarily activated the microwave modules during drying to determine: (A) whether the mAb is initially damaged when microwave radiation is started, or (B) if particle formation inversely correlates with residual water content. We concentrated on analyzing subvisible particles, as they proved to be a reliable degradation indicator in our previous experiments. First, using lyophilization cycle P3 (Table 2), microwaves were applied either in the first 5 h of the drying phase (Figure 4A) or toward the end of the drying process (Figure 4B). When microwave radiation was applied initially, subvisible particle counts were at the placebo level regardless of the mAb concentration (Figure 4C). However, we observed a significant increase in protein aggregates in F5 compared to F1 and the placebo formulation when microwaves were applied late in the drying process. The reason why the number of small subvisible particles, between 1 µm and 10 µm, increased in the placebo formulation as well, when microwaves were applied later in the process, merits further study. 

Based on these findings, we conducted four additional runs and subsequently extended the microwave radiation time. The microwave modules were activated at the beginning of the drying process and ran continuously for 6 h, 8 h, 10 h, and 13 h (Figure S3A–D). To prevent sample overheating during MFD, cycles with 10 and 13 h microwave runtime were conducted using process P4 (Table 2), while runs with 6 and 8 h of microwave radiation used process P3. This resulted in differences in product temperature across different runs (Figure 4D); however, the residual moisture and associated glass transition temperature of the cakes was similar for F5 (Figure S4). Due to the aggressive drying conditions, scanning electron microscopy revealed a microcollapsed morphology in F1 for all processes, while cellular pore structures were observed for F5 (Figure S5). Moreover, the point of termination of microwave radiation is clearly visible in all curves (Figure 4D).

Although product temperature during drying did not increase with longer microwave runtime due to the chosen settings (Figure 4D), aggregate formation clearly correlated with radiation time for F5 (Figure 4E). While the low-concentration formulation F1 equaled the placebo irrespective of runtime, we observed a gradual increase in subvisible particle counts in the high-concentration mAb formulation F5.

Since ice exhibits a low dielectric loss factor [21], microwaves most likely excite highly polarizable unfrozen water [30] and other excitable formulation components. We therefore hypothesize that protein preservation occurs as long as heat may be dissipated throughout the matrix; otherwise, damage takes place. As the dielectric properties of formulations change during drying [31], the very late stage of the drying process is considered particularly problematic concerning the physicochemical stability of active compounds [21]. However, our studies uncovered that high-concentration mAb formulations are susceptible to degradation much earlier; this occurs after just a few hours of drying when sublimation is still high.

### 3.5. Effect of Residual Moisture, Cooling, and the Source of Energy

The previous experiments raised the question of whether there is a potential tipping point during the MFD of highly concentrated protein formulations that leads to aggregation. To explore this, we conventionally lyophilized F5 (Process P2) and used the dried cakes to conduct experiments in a microwave oven. 

**Figure 5:**
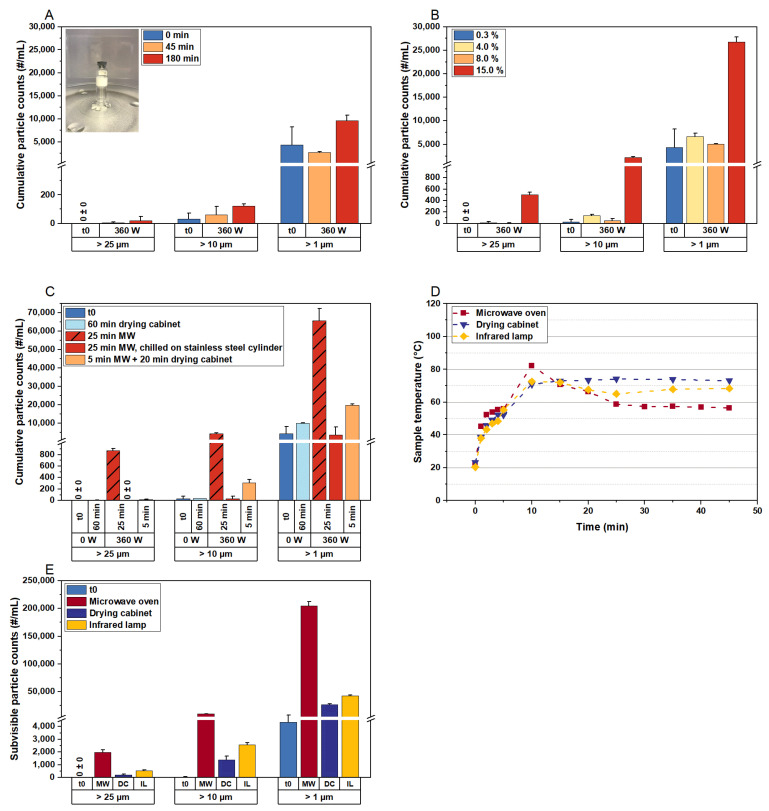
The impact of microwave radiation on protein aggregate formation in lyophilized formulation F5. Initial subvisible particle counts (t0) were determined immediately after conventional freeze-drying. (**A**) Samples were exposed to 360 W for different durations, without chilling during exposure to microwave radiation. A polymeric vial was used as a spacer to insulate the samples from the rotating glass plate in the microwave oven. This setup was used for the following experiments, with the data shown in (**B**–**E**). (**B**) The formation of subvisible particles with increased residual moisture. The residual moisture content of 15% was adjusted in all processed samples shown in (**C**–**E**). (**C**) Comparison of convective heat transfer and microwave heating, with the drying cabinet temperature set to 80 °C. To mimic freeze-drying conditions, the vial was placed on a precooled stainless steel cylinder inside the microwave oven (red bars, without pattern). (**D**,**E**) Lyophilizates were subjected to three different energy sources. (**D**) The temperature within the cakes and (**E**) the corresponding formation of protein aggregates. The subvisible particle data represent the mean values of technical duplicates per vial ± standard deviation. MW, microwave irradiation.

Initially, we applied 360 W to the lyophilizates without cooling the vials during the experiment, using a polymeric vial for the insulation of the samples from the glass plate (Figure 5A). No relevant increase in subvisible particle counts was detected even after 180 min of irradiation. These results led us to conclude that the dried cake does not represent a worst-case scenario for aggregate formation during MFD, as the antibody is immobilized in a rigid matrix.

We then increased the residual moisture in the cakes to examine whether the moisture content and associated mobility comprise a dominant factor affecting aggregation. Different moisture levels were adapted according to the technique from [32], and we observed a significant increase in subvisible particle counts at an intermediate moisture level of 15% (m/m) (Figure 5B), which corresponds to the typical moisture content at the end of primary drying in a CFD process [33]. This confirmed our hypothesis that a certain degree of residual water and anti-plasticization is a prerequisite for aggregate formation.

Considering these findings, we adjusted the residual moisture to 15% (m/m) for all subsequent samples (except t0) and compared subvisible particle counts following different treatments (Figure 5C). Samples exposed to convective heat transfer at 80 °C in a drying cabinet showed low particle counts (light-blue bars). To mimic freeze-dryer shelf conditions, we placed a sample on a precooled stainless steel cylinder during microwave irradiation. Interestingly, cooling the sample protected the mAb from degradation, as no increase in protein aggregates was detected after 25 min in the microwave oven (red bars), contrasting with the uncooled sample that exhibited significant particle counts (hatched red bars). In another treatment, the sample was placed in the microwave oven for 5 min, followed by 20 min in the drying cabinet, resulting in slightly increased particle counts compared to convective heat application alone (orange bars).

To investigate differences in heating with microwaves versus other heat transfer methods, we exposed samples to microwaves, infrared radiation, and convective heat in a drying cabinet, aligning the temperature profiles for comparability (Figure 5D). We observed a significant increase in subvisible particle counts following microwave irradiation compared to other heating methods (Figure 5E), concluding that microwave radiation directly excites polar groups in the antibody structure, leading to protein aggregate formation. 

It has been demonstrated that the intermediate, rubbery state during drying processes, characterized by considerable moisture content and low glass transition temperatures (T_g_′), is the most detrimental phase for protein stability [34]. Increased concentrations of the protein in the viscous glassy matrix still allowing for notable mobility, as water is not sufficiently removed, make protein degradation more likely. This is consistent with our findings in MFD. We found that cooling the sample can provide some protection for the mAb (Figure 5C). However, this presents a deadlock in the drying process, as complete drying while maintaining cold temperatures is unattainable. Moreover, the need for cooling to preserve protein stability prevents the full exploitation of MFD technology. Our findings show that high product temperatures are only problematic for the stability of the mAb when microwave radiation is applied.

The preservation of a protein’s native structure during lyophilization via adding an adequate ratio of lyoprotectant has been well documented [4,29]. With growing interest in high-concentration mAb formulations [35], e.g., for subcutaneous injections, high disaccharide concentrations are often required, and the reconstitution time is directly influenced by the sugar:protein ratio [36]. Our studies revealed the importance of sugar:protein ratios regarding stabilization in MFD technology. MFD is a competitive technology for low-concentration protein formulations; however, for high-concentration mAb formulations, water replacement via the classical approach [4,29] was insufficient. Additional research is required to determine whether an optimized sugar:protein ratio or other formulation compositions could provide enhanced protection for high-concentration protein formulations during microwave-assisted freeze-drying.

## 4. Conclusions

These studies are connected to previous work on a novel microwave-assisted freeze-drying setup [17] and provide a first design space for the use of this technology. While the applicability of MFD for low-concentration protein formulations is reaffirmed, we observed particle formation with high-concentration antibody formulations, which were not observed for conventional freeze-drying controls. We demonstrated that microwaves directly interact with the active pharmaceutical ingredient (API), and the higher the API concentration, the more protein could be excited by the microwaves. This interaction resulted in decreased physical stability in the investigated high-concentration antibody formulations, manifesting as the formation of subvisible protein aggregates. Additionally, we showed that particle formation does not occur immediately after starting MFD, but during the intermediate drying phase. However, since the collapse temperature significantly increases with higher protein concentrations, reduced drying times for high-concentration protein formulations can be also achieved using aggressive CFD conditions [25]. In this configuration, the potential benefit of MFD regarding reduced process times is anyway limited. Based on our findings, we believe that MFD technology is particularly beneficial for low-concentration formulations requiring substantial amounts of glass-forming excipients, which normally limit time savings in CFD. Here, one could, of course, envision the fast, mass production of, e.g., vaccines that typically contain a relatively low-to-very-low amount of protein or another antigen. Furthermore, modern RNA-based products and vaccines, as well as virus and virus-like particle (VLP) formulations, etc., also containing a rather-low-to-very-low total amount of active ingredient in the matrix and can potentially benefit from MFD.

## Figures and Tables

**Table 1:** Formulations investigated in the study.

Formulation Number	Protein Conc. (g/L)			Sucrose (%)	Trehalose (%)	Mannitol (%)	Methionine (mM)	PS 20 (%)	pH
	LMU1	LMU2	G-CSF						
F1	10			8				0.040	5.5
F2	30			6				0.040	5.5
F3	50			4				0.040	5.5
F4	70			2				0.040	5.5
F5	100			8				0.040	5.5
F6		21			1.9			0.009	6.0
F7			0.5	1		4	20	0.010	4.0

Conc., concentration; PS 20, polysorbate 20.

**Table 2:** Applied drying protocols in the study.

Drying Process	Step	T_s_ (°C)	P_c_ (mbar)	Hold Time (h)	Ramp Toward Step (K/min)	MW Application (W)
P1	1	−15	0.05	*	1.0	2 × 90 **
	2	30	0.05	6	1.0	2 × 90 **/†
P2	1	30	0.05	*	0.2	
P3	1	30	0.05	*	0.2	2 × 90 ‡
P4	1	10	0.05	*	0.2	2 × 90 §
	2	30	0.05	4	1.0	-

* Maintained until Pirani signal equaled capacitance, and mass spectrometer revealed water vapor concentration c_H2O_ < 10%. ** In case of MFD. † Applied continuously until the shelf temperature reached 0 °C to not overheat the samples. ‡ Microwave module was stopped after 5 h, 6 h, and 8 h respectively. In case of MFD of F7, 2 × 90 W were applied until Pirani signal equaled capacitance sensor output, and mass spectrometer revealed water vapor concentration c_H2O_ < 10%. § Microwave module was stopped after 10 h and 13 h, respectively. MW, microwave.

## Data Availability

The data are contained within the article.

## Supplementary Materials

The following supporting information can be downloaded at: https://www.mdpi.com/article/10.3390/pharmaceutics15122783/s1, Figure S1: Representative SEM pictures of the top and bottom of the lyophilizates of F1–F7 after MFD, captured at 175-fold magnification. Figure S2: Readouts from drying processes of F5. T_s_ represents the shelf temperature; the chamber pressure is monitored via a Pirani gauge (Pirani) and capacitance gauge (Capacitance); T_p_ refers to the readouts of the temperature sensors. (A) Formulation F5 underwent MFD according to process P1. (B) To mimic the temperature profiles of (A), process P2 was applied to F5 (i.e., without microwave radiation). Figure S3: Readouts from the MFD processes with varying microwave run times. Ts represents the shelf temperature; the chamber pressure is monitored via a Pirani gauge (Pirani) and capacitance gauge (Capacitance); Tp refers to the readouts of the fiberoptic temperature sensors. Microwave modules were manually started (MW on) and automatically terminated (MW off) after (A) 6 h, (B) 8 h, (C) 10 h, and (D) 13 h. Figure S4: Solid-state properties of F1 and F5 lyophilizates after various microwave run times. Glass transition temperature (bars) and residual moisture (symbols) were measured immediately following lyophilization. The values represent the means (*n* = 3, except for T_g_ of F1 6 h and 8 h where *n* = 1) ± standard deviation. Figure S5: Representative SEM pictures of the top and bottom of the lyophilizates of (A) F1 and (B) F5 after different microwave runtimes. Images were captured at 175-fold magnification.
