# Supplementary material for: Microwave-Assisted Freeze–Drying: Impact of Microwave Radiation on the Quality of High-Concentration Antibody Formulations
Source: Pharmaceutics. 2023 Dec 15;15(12):2783. doi: 10.3390/pharmaceutics15122783 (PMC10747838; doi:10.3390/pharmaceutics15122783)
Supplement: Supplementary file 1 [file pharmaceutics-15-02783-s001.zip › Supplementary materials.pdf]

pharmaceutics

MDPI

Microwave-Assisted Freeze-Drying: Impact of Microwave Radiation on the Quality of High-Concentration Antibody Formulations

Nicole Härdter, Raimund Geidobler, Ingo Presser and Gerhard Winter

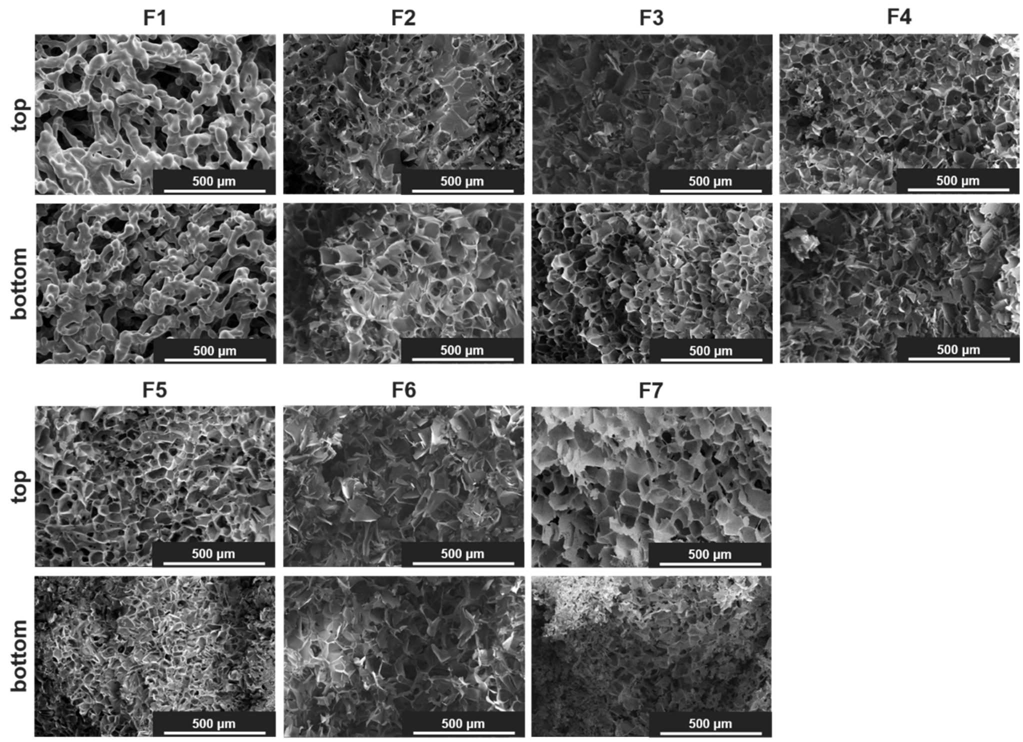

Figure S1. Representative SEM pictures of the top and bottom of the lyophilizates of F1–F7 after MFD, captured at 175-fold magnification.

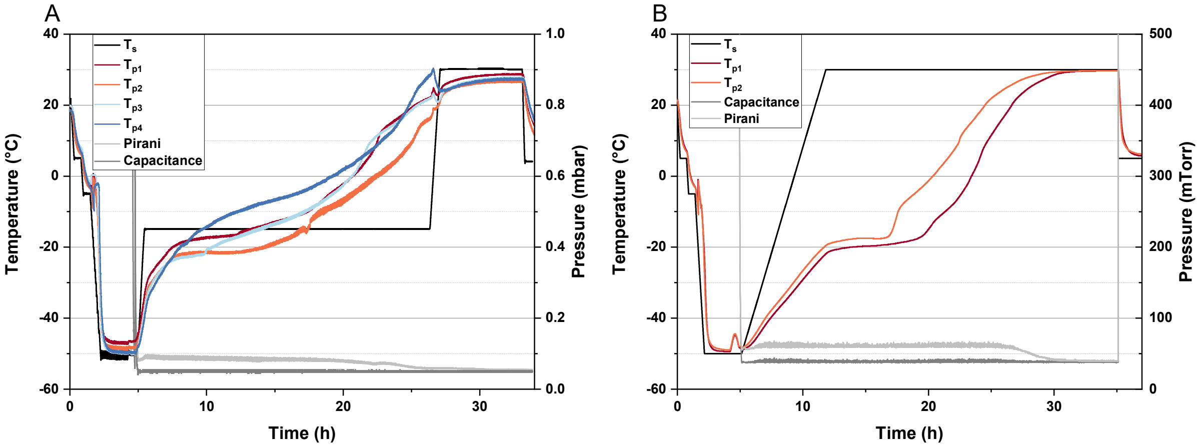

Figure S2. Readouts from drying processes of F5. T_s represents the shelf temperature; the chamber pressure is monitored via a Pirani gauge (Pirani) and capacitance gauge (Capacitance); T_P refers to the readouts of the temperature sensors. (A) Formulation F5 underwent MFD according to process P1. (B) To mimic the temperature profiles of (A), process P2 was applied to F5 (i.e., without microwave radiation).

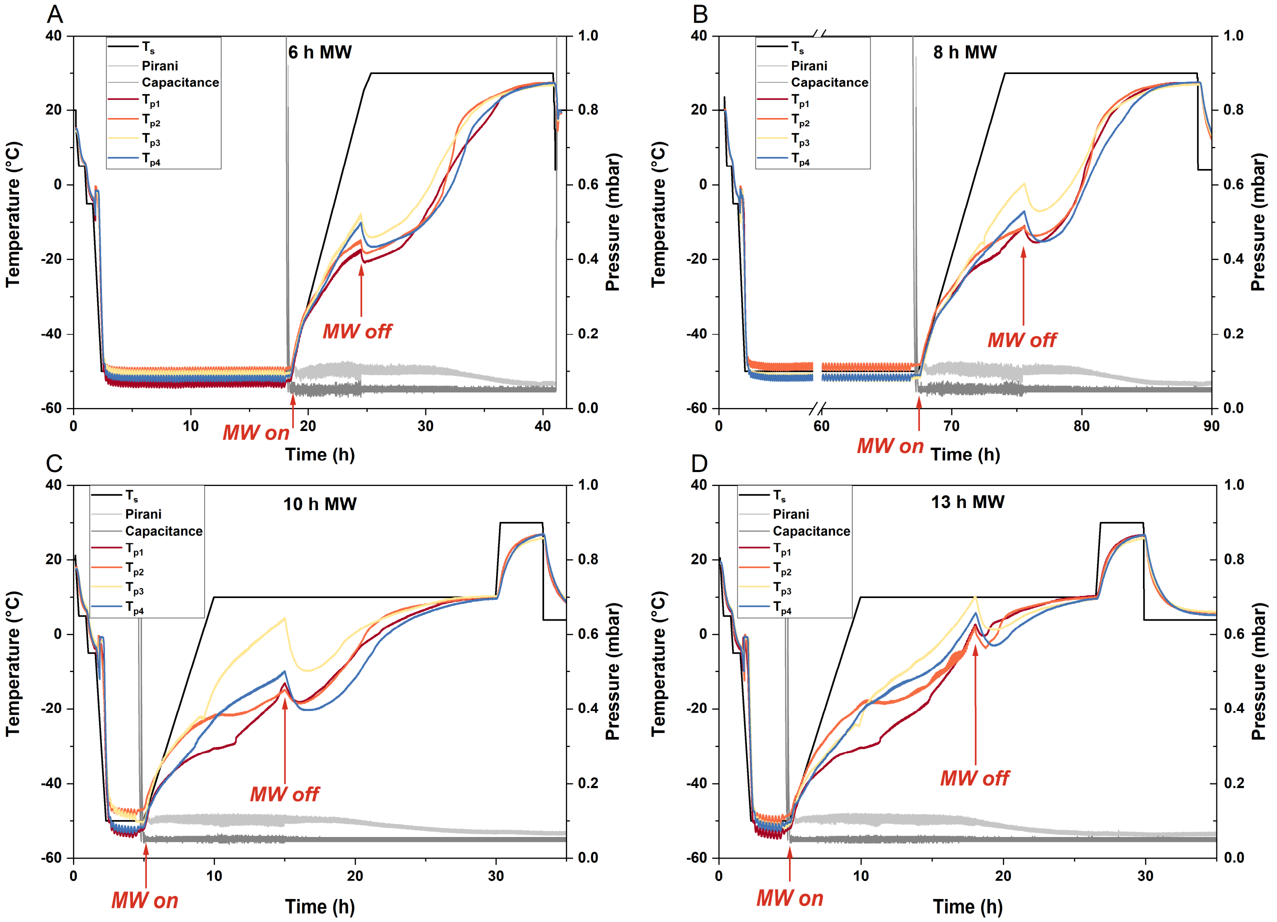

Figure S3. Readouts from the MFD processes with varying microwave run times. T_s represents the shelf temperature; the chamber pressure is monitored via a Pirani gauge (Pirani) and capacitance gauge (Capacitance); T_p refers to the readouts of the fiber optic temperature sensors. Microwave modules were manually started (MW on) and automatically terminated (MW off) after (A) 6 hours, (B) 8 hours, (C) 10 hours, and (D) 13 hours. MW, microwave irradiation.

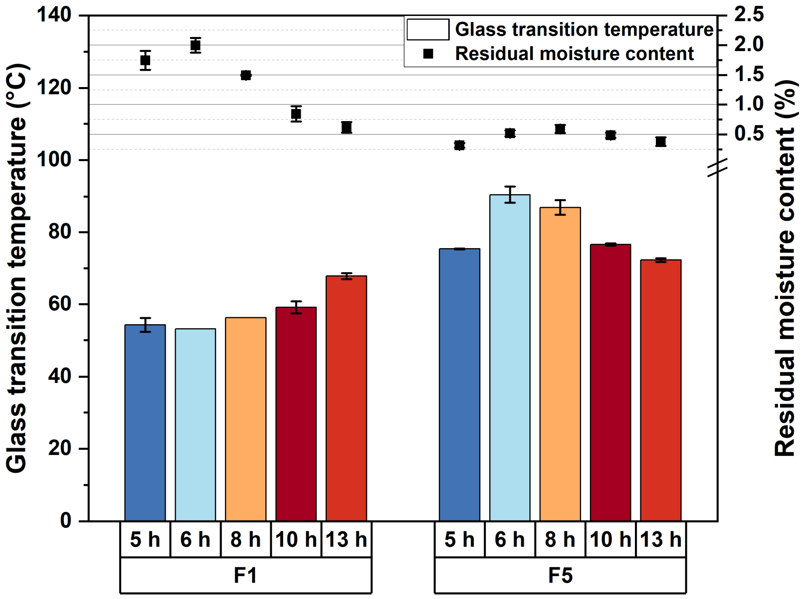

Figure S4. Solid-state properties of F1 and F5 lyophilizates after cycles with various microwave run times. Glass transition temperature (bars) and residual moisture (symbols) were measured immediately following lyophilization. The values represent the means (n = 3, except for T_g of F1 6h and 8h where n = 1) ± standard deviation.

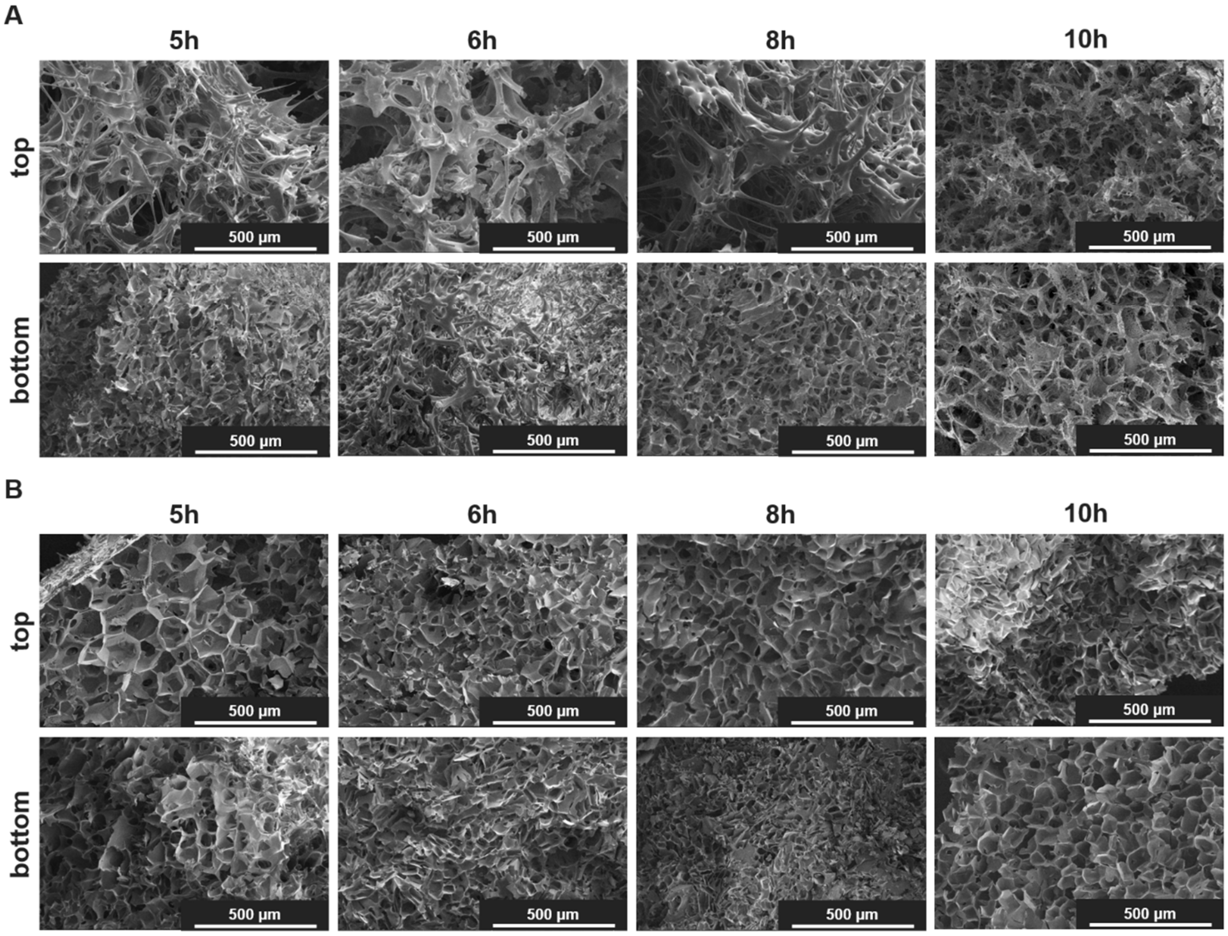

Figure S5. Representative SEM pictures of the top and bottom of the lyophilizates of (A) F1 and (B) F5 after different microwave runtimes. Images were captured at 175-fold magnification.
